# Subclass Mapping: Identifying Common Subtypes in Independent Disease Data Sets

**DOI:** 10.1371/journal.pone.0001195

**Published:** 2007-11-21

**Authors:** Yujin Hoshida, Jean-Philippe Brunet, Pablo Tamayo, Todd R. Golub, Jill P. Mesirov

**Affiliations:** 1 The Eli and Edythe L. Broad Institute, Massachusetts Institute of Technology and Harvard University, Cambridge, Massachusetts, United States of America; 2 Pediatric Oncology, Dana-Farber Cancer Institute, Boston, Massachusetts, United States of America; South African National Bioinformatics Institute, South Africa

## Abstract

Whole genome expression profiles are widely used to discover molecular subtypes of diseases. A remaining challenge is to identify the correspondence or commonality of subtypes found in multiple, independent data sets generated on various platforms. While model-based supervised learning is often used to make these connections, the models can be biased to the training data set and thus miss inherent, relevant substructure in the test data. Here we describe an unsupervised subclass mapping method (SubMap), which reveals common subtypes between independent data sets. The subtypes within a data set can be determined by unsupervised clustering or given by predetermined phenotypes before applying SubMap. We define a measure of correspondence for subtypes and evaluate its significance building on our previous work on gene set enrichment analysis. The strength of the SubMap method is that it does not impose the structure of one data set upon another, but rather uses a bi-directional approach to highlight the common substructures in both. We show how this method can reveal the correspondence between several cancer-related data sets. Notably, it identifies common subtypes of breast cancer associated with estrogen receptor status, and a subgroup of lymphoma patients who share similar survival patterns, thus improving the accuracy of a clinical outcome predictor.

## Introduction

DNA microarray-based whole genome expression profiling is subject to poor reproducibility of discovered molecular disease subtypes and can lead to biomarkers that do not generalize [1]. This problem arises from various technical and biological sources including platform differences [2], and has been a major obstacle to moving microarrays into the clinic as a tool to uncover as yet unrecognized disease subtypes.

Comparison and integration of the molecular disease subtypes, independently defined in different data sets, has been a highly challenging problem. Subtypes are often based on subtle differences in gene expression, which can be dominated by the measurement variation between different experiments and/or platforms. A widely used method to connect such independent data sets, supervised learning, does not completely solve this problem. Subtype models depend on one particular “training” data set with its own platform-specific data structure. This structure may not be present in new “test” data sets.

Here we describe subclass mapping (SubMap), an unsupervised method that reveals common subtypes observed in independent data sets without relying on, or being biased by, one model data set. By bi-directionally evaluating association of predetermined subtypes between independent data sets, our method identifies more reliable molecular disease subtypes and subpopulations of cohorts that share similar clinical behavior. These results indicate the great potential of this method to maximize the use of the vast amount of accumulating genomics data and to improve current clinical practice through the development of better diagnostics and therapeutics.

## Results

### Overview of Subclass mapping (SubMap)

SubMap is an unsupervised method, which estimates the significance of an association between subclasses observed in two independent data sets. The subclass labels are predetermined as manually assigned phenotypes or by clustering prior to the application of the SubMap algorithm (i.e., the SubMap algorithm does not assign a *de novo* class label to each sample). Because the subclass correspondence is evaluated for all pairs of subclasses, one subclass drawn from each data set, the number of subclasses or subtypes in the two data sets does not need to be the same or even similar.

**Figure 1:**
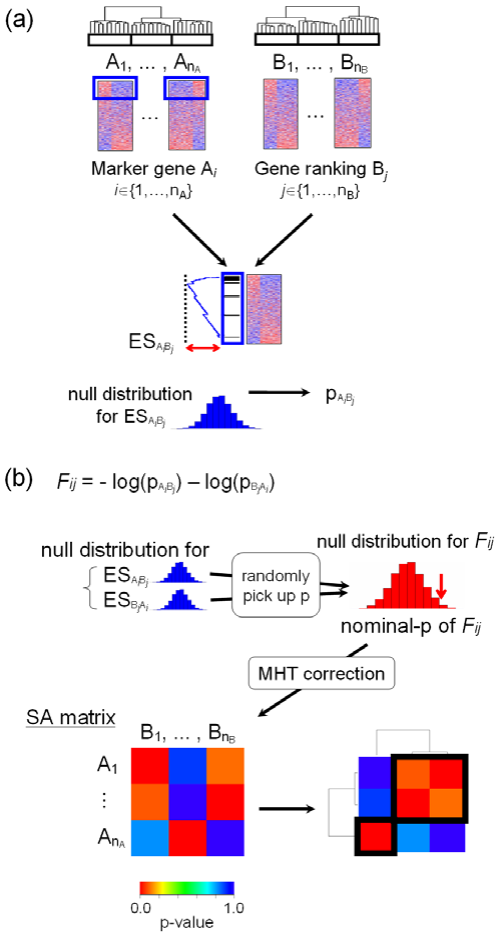
Subclass mapping (SubMap) methodology. Two independent data sets, A and B, are clustered separately, compared and integrated. (a) Candidate subclasses are defined by clustering A and B (predetermined phenotype can also be used). Marker genes of each candidate subclass in A (A*_i_*) are selected, and mapped onto a gene list ranked according to their differential expression with respect to a subclass of B (B*_j_*). Their over-representation at the top of the ranking is evaluated using the enrichment score (ES_A*i*B*j*_), and significance is assessed as a nominal p-value, p_A*i*B*j*_, by randomly permuting sample class labels in B. This process is repeated by interchanging the role of A and B to compute ES_B*j*A*i*_ and p_B*j*A*i*_. (b) Mutual enrichment information, p_A*i*B*j*_ and p_B*j*A*i*_, are combined using the Fisher inverse chi-square statistic, *F_ij_*. Its significance is estimated based on a null distribution for the *F_ij_* generated by randomly picking the nominal-p from corresponding null distributions for ES_A*i*B*j*_ and ES_B*j*A*i*_. After multiple hypothesis testing (MHT) correction, p-values for *F_ij_* are summarized in the subclass association (SA) matrix. Clustering of the SA matrix reveals subclasses common to A and B.

The mapping information obtained by SubMap can be used to reveal general subclasses common to both data sets and thus expose the likelihood that they share the same or similar underlying biological property. Details of our approach are described in the Materials and Methods section and Figure 1. Here we review the three key steps in the SubMap method:

#### Step 1: Measure similarity between subclasses

We start with two independent data sets, A and B, with candidate subclasses independently determined in both. For simplicity, suppose we have two candidate subclasses in each data set: A1 and A2 in data set A; B1 and B2 in data set B. We define a set of marker genes, *marker*(A1) for subclass A1, and similarly for A2, using any suitable subclass discrimination metric. Using the same metric, genes in data set B are rank-ordered according to their correlation with B1 vs. B2 to yield a gene list, *ranking*(B1). Association between A1 and B1 is evaluated by quantifying the over-representation of *marker(*A1) in the up-regulated end of the list *ranking*(B1) using Gene Set Enrichment Analysis (GSEA) as described in [3]. The latter provides both an enrichment score (ES) and a corresponding nominal p-value by means of an empirical permutation test. We repeat this process, interchanging the roles of A1 and B1, to compute a nominal p-value for the enrichment of *marker*(B1) in *ranking*(A1). We can generalize this process straightforwardly when there are more than two candidate subclasses in either data set and compute a pair of nominal p-values for every possible pair-wise combination of subclasses. Thus if there are *n_A_* subclasses in A and *n_B_* subclasses in B, then we have mutual association information for each of the n_A_*n_B_ pairs of candidate subclasses from the two independent data sets.

#### Step 2: Estimate significance of association between subclasses

To summarize and estimate the significance of the mutual association information, i.e., the pair of nominal p-values for the ES, we use the Fisher inverse chi-square statistic, *F* [4]. To estimate a nominal p-value for *F*, we generate an appropriate null distribution by randomly picking ES scores from the null distributions corresponding to each direction of the enrichment analysis (e.g., *marker*(A1) in *ranking*(B1) and *marker*(B1) in *ranking*(A1)) and generating the corresponding Fisher inverse chi-square statistic.

#### Step 3: Construct and cluster subclass association matrix

We use a Bonferonni adjustment to account for multiple hypotheses testing, and summarize the adjusted p-values in a matrix called the subclass association matrix (SA matrix). Two-way clustering of this matrix reveals general subclasses common to these data sets.

We applied our method to four pairs of publicly available data sets (Table 1). We first validated our method by correctly recovering expected associations of subclasses using a pair of data sets comprised of multiple tissue types (Example 1). Next we analyzed diffuse large B cell lymphoma (DLBCL) data sets to show the superiority of our method to standard supervised prediction methods (Example 2). We then applied our method to breast cancer data sets to identify a common subtype we found to be associated with the estrogen receptor status (Example 3). The final example shows that our method has the potential to improve the performance of molecular marker-based patient outcome prediction (Example 4). We note that all of these analyses were performed on data sets acquired on a wide variety of DNA microarray platforms.

**Table 1:** Data sets

Group	Data set	No. of samples	Type of microarray	Platform description	GEO platform ID	reference
1. Multiple tissue types (breast, lung, prostate, colon)	Multi-A	103	1-channel	HG-U95A*	GPL91	[5], (a)
	Multi-B	32	1-channel	HuGeneFL, Hu35k-A*	GPL80, 98	[6], (a)
2. Diffuse large B cell lymphoma	DLBCL-A	141	1-channel	HG-U133A*	GPL96	[7], (a)
	DLBCL-B	180	2-channel	Lymphochip	N/A	[8], (b)
3. Breast cancer	Breast-A	98	2-channel	Hu25K**	N/A	[11], (c)
	Breast-B	49	1-channel	HuGeneFL*	GPL80	[12], (a)
4. Diffuse large B cell lymphoma (with survival data)	DLBCL-C	58	1-channel	HuGeneFL*	GPL80	[14], (a)
	DLBCL-D	129	1-channel	HG-U133A*	GPL96	[7], (a)

1-channel: sample RNA is labeled by single dye, 2-channel: sample and reference RNA are labeled by different

dyes and competitively hybridized.

GEO: National Center for Biotechnology Information's Gene Expression Omnibus (www.ncbi.nlm.nih.gov/geo/),

GPL and GSE are accession number for miceroarray platform and gene set, respectively, depositted in th GEO.

(a) http://www.broad.mit.edu/cgi-bin/cancer/datasets.cgix

(b) http://llmpp.nih.gov/DLBCL/

(c) http://www.rii.com/publications/default.htm

Microarrays manufactured by *Affymetrix (Santa Clara, CA) or ** Agilent Technologies (Palo Alto, CA)

### Example 1. Multiple tissue types

**Figure 2:**
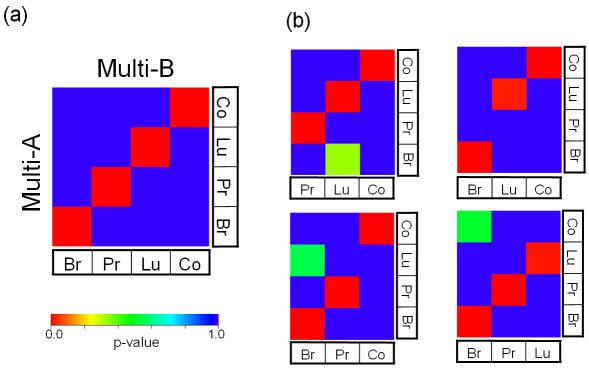
Example 1: Multiple tissue types. (a) SubMap was applied to two data sets, Multi-A and Multi-B, containing multiple tissue types: breast (Br), prostate (Pr), lung (Lu), and colon (Co). Bonferroni-corrected p-values for breast, prostate, lung, and colon tissues were 0.002, 0.002, 0.002, and 0.002, respectively. (b) Each tissue type in Multi-B was removed before applying SubMap. Only subsets of the same tissue type were significantly associated (Bonferoni-corrected p<0.05). The p-values for “Multi-A-Br and Multi-B-Lu (left-upper)”, “Multi-A-Lu and Multi-B-Br (left-bottom)”, and “Multi-A-Co and Multi-B-Br (right-bottom)” are 0.330, 0.547, and 0.517, respectively

For a straightforward validation of subclass mapping, we used two data sets of multiple normal tissue types acquired on two different generations of the Affymetrix GeneChip oligonucleotide microarray platform, Multi-A [5] and Multi-B [6]. This represents the case where subtypes are determined by ancillary phenotype information rather than “discovered” by clustering. Both data sets include four distinct tissue types, i.e., breast, prostate, lung, and colon. The only cross-dataset pairs of subtypes showing significant association corresponded to the same tissue type (Figure 2a).

In actual biological or clinical data sets that nominally include the same or similar samples, the entire range of sample diversity in one data set may not be represented in the other data sets, i.e., a subclass in one data set may not be represented in others. To assess whether such a situation would lead to false positive associations, we removed the samples corresponding to one tissue type in Multi-B and re-ran SubMap with the Multi-A (Figure 2b). Importantly, only subsets of the same tissue type were significantly associated, and there were no false positive calls.

We next investigated whether the choice of the number of marker genes to be mapped might affect the result of SubMap. The number of strongly differentially expressed genes for cross-dataset subclass pairs might differ. Since we are using the same number of marker genes for all comparisons we sought to confirm that the enrichment score was robust. We believed this would be the case based on the robustness of the GSEA score. We tested the effect of using 20, 50, and 200 ranked marker genes from a list of differentially expressed genes, as they represent the number of markers commonly used in classification and prediction studies for microarray data, as well as the level at which we might expect to begin to see noise. The resulting observed significance calls were quite stable for pairs of same tissue type irrespective of the number of the marker genes used (Figure S1).

### Example 2. Diffuse large B cell lymphoma: comparison with other methods

Here we show the superiority of SubMap in directly highlighting all three of the corresponding, common subtypes in two independent Diffuse Large B-Cell Lymphoma (DLBCL) data sets. We first compare with our previous work in [7] where two of the three common subtypes could only be associated after removing the third. Importantly, we also show how supervised approaches, building a model from one data set to identify the subtypes in the other, also fail to identify all three shared subtypes.

We analyzed two DLBCL data sets, DLBCL-A [7] and DLBCL-B [8]. The data sets were generated using one-channel oligonucleotide microarrays and 2-channel custom cDNA microarrays, respectively. In previous work we found three robust subtypes in both DLBCL-A and DLBCL-B using resampling-based multiple clustering trials and three different clustering algorithms [7], [9]. The three clustered subtypes were designated as “oxidative phosphorylation (OxPhos)”, “B-cell response (BCR)”, and “host response (HR)” according to relevant molecular mechanisms.

**Figure 3:**
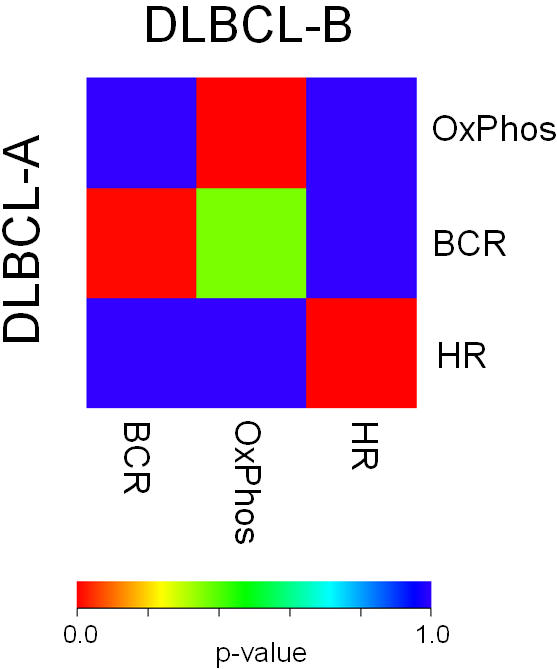
Example 2: Common subtypes of Diffuse Large B-cell Lymphoma (DLBCL). SubMap was applied for three subclasses of DLBCL pre-determined in DLBCL-A and DLBCL-B data sets. Bonferroni-corrected p-values for “oxidative phosphorylation (OxPhos)”, “B-cell response (BCR)”, and “host response (HR)” subtypes were 0.008, 0.001, and 0.001, respectively. The association for the pair of DLBCL-A-BCR and DLBCL-B-OxPhos was not significant (p = 0.362).

In this precedent analysis, we associated these subtypes between the data sets by assessing the overlap of each subtype's marker genes in both data sets one-by-one using the Fisher test for a 2×2 table. However, unlike SubMap, this approach is highly sensitive to the criteria for marker gene selection and the total number of genes common to both platforms. Notably, initially only the HR clusters from the two datasets showed a significant association, and only after their removal could we identify the significant association for the OxPhos and BCR subtypes [7]. However, our SubMap method immediately and automatically recovered the three-subclass structure common to both data sets without removing any samples (Figure 3). This is likely due to the method's ability to capture subtle, but reproducible, subclass associations by incorporating bi-directional marker gene enrichment information.

Next we compared SubMap with supervised methods for their ability to identify similar subtypes from distinct data sets using DLBCL-A and DLBCL-B. Supervised learning methods are widely used to associate two independent data sets; the model of the subtype is defined in a “training” data set, and assessed in a “test” data set. We employed two standard supervised methods, k-nearest neighbors (k = 7, and 10 marker genes) and support vector machines (using all genes in common) and interchanged the roles of the two sets as train and test (details are described in the Materials and Methods). Both the k-NN and SVM predictors failed to reliably predict all three DLBCL subtypes. When the DLBCL-B was used to train the model, only the HR clusters were associated by prediction (accuracy≥90%). When the DLBCL-A was used to train the model, only the BCR clusters were associated by the SVM classifier (accuracy≥90%), and none by the k-NN predictor (all accuracies≤51%). (See Table S1 for full results). In addition, we tested a recently reported method, called clusterRepro [10]. This method assumes a model of subclassification in one data set, and evaluates it in another data set. Again we only observed significant reproducibility (accuracy≥90%) for the HR cluster, in this case when DLBCL-A was used as the model set (Table S2).

In this example we see the difficulty of identifying common subclasses and how SubMap outperforms four other approaches.

### Example 3. Breast cancer: estrogen receptor status

We next compared SubMap method with another more straightforward approach, i.e., to merge the two independent data sets into a single data set, and and then to run clustering to find subtypes observed in the combined data. To this end, we employed two breast cancer data sets, Breast-A [11] and Breast-B [12] generated using one-channel oligonucleotide and two-channel microarrays, respectively. Hierarchical clustering, after simply merging the two data sets, only revealed the dominant experiment-of-origin-specific structure despite several attempts of normalization (Figure S2), whereas SubMap identified common subtypes associated with a key molecular determinant of a disease as described below.

**Figure 4:**
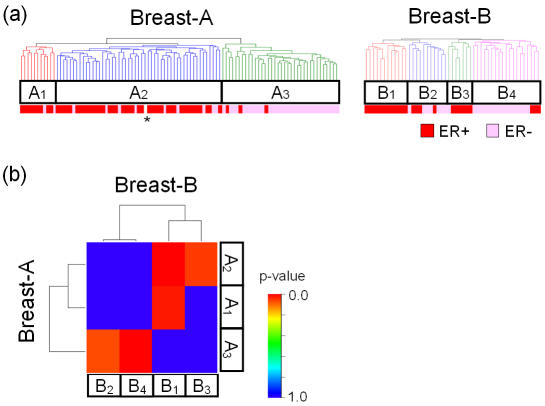
Example 3: Common subtypes of breast cancer associated with estrogen receptor (ER) status. (a) Candidate subclass labels were assigned using hierarchical clustering in Breast-A and Breast-B data sets independently. (b) Subclass association (SA) matrix for Breast-A and Breast-B. Bonferroni-corrected p-values for the combinations of “A_1_ and B_2_”, “A_1_ and B_4_“, “A_2_ and B_1_”, “A_3_ and B_1_”, and “A_3_ and B_3_” were 0.070, 0.002, 0.023, 0.001, and 0.055, respectively (FDR-corrected p-values of 0.014, 0.001, 0.008, 0.001, and 0.014, respectively). *: ER status is missing for one case.

Using hierarchical clustering as described in Materials and Methods, we identified three (A_1_, A_2_, and A_3_) and four (B_1_, B_2_, B_3_, and B_4_) candidate subclasses in Breast-A and Breast-B, respectively (Figure 4a) as described in the Materials and Methods section. SubMap revealed the common subtypes as two sets of significant associations, “A_1_ ∪ A_2_ and B_1_ ∪ B_3_“ and “A_3_ and B_2_ ∪ B_4_“ (Figure 4b). We note the dominant substructure (i.e., first splitting in the dendrogram in Figure 4a) in Breast-B is B_1_ ∪ B_2_ and B_3_ ∪ B_4_. Thus, the subgrouping of samples from Breast-B that associate with Breast-A might not be evident by looking at Breast-B alone.

Both data sets have immunohistochemistry data of estrogen receptor (ER) status, which is known to play important role in breast cancer biology, e.g., BRCA1 mutation, lymphocytic infiltration, and early occurrence of distant metastasis [11], [13]. In fact, this was the only sample-phenotype information common to both data sets. Thus, we sought to evaluate if the common subtypes obtained by our method captured this ancillary phenotypic information. We found that the common subtypes defined by SubMap were significantly associated with the ER status in each data set (Breast-A: ER was positive in 50/61 cases of A_1_ ∪ A_2_, and 3/36 cases of A_3_. p = 4.2e-13. Breast-B: ER was positive in 18/19 cases of B_1_ ∪ B_3_, and 7/23 cases of B_2_ ∪ B_4_. p = 8.0e-7. Fisher's exact test). This result leads us to speculate that ER status may indeed be a significant factor in determining the subclasses and subclass associations.

To investigate this hypothesis further, we evaluated the expression status of ER signaling-related genes in the common subtypes to confirm that the subtypes are relevant to the biology of ER signaling. In both input data sets, we ranked genes by the extent of their up-regulation in the subtypes with more ER positive cases (A_1_ ∪ A_2_ and B_1_ ∪ B_3_), and evaluated the over-representation of the ER-related gene set (Table S3) using Gene Set Enrichment Analysis [3]. The ER-related gene set was significantly over-expressed with p-values of 0.033 and 0.010 in A_1_ ∪ A_2_ within Breast-A and B_1_ ∪ B_3_ within Breast-B, respectively.

The association between A_1_ and B_3_ was not statistically significant (Figure 4b). In fact, the number of cases with lymph node metastasis in B_1_ (8/12, 67%) is larger than that of B_3_ (2/7, 29%). Although this difference is not statistically significant (p = 0.170, Fisher's exact test), this might suggest the existence of some biological heterogeneity among the ER-positive subclasses, and cause such “step-like” shape.

### Example 4. Diffuse large B-cell lymphoma: patient survival

In this final example, we applied SubMap to see if identifying the corresponding subtypes in two diffuse large B-cell lymphoma data sets would enable us to distinguish subpopulations relevant to patient survival. We analyzed two independent DLBCL data sets, DLBCL-C (n = 58) [14] and DLBCL-D (n = 129) [7]. DLBCL-D contains the samples in DLBCL-A from Example 2 for which survival information was available. DLBCL-C is a third independent data set. These data sets were generated using different generations of the Affymetrix GeneChip oligonucleotide microarray.

**Figure 5:**
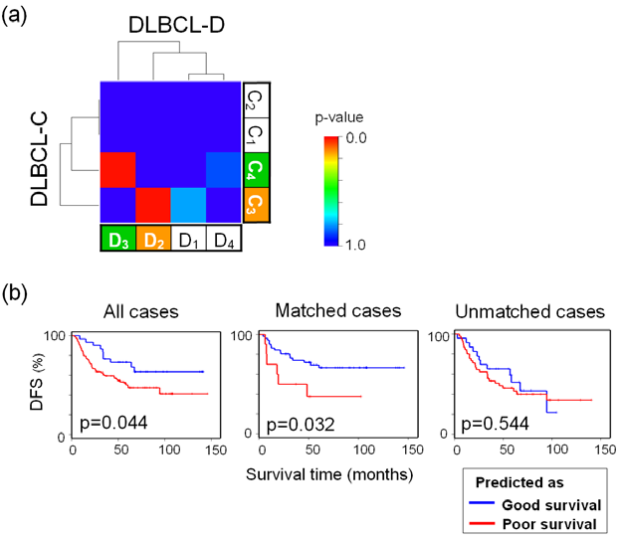
Example 4: Survival prediction in Diffuse Large B-cell lymphoma (DLBCL) data sets. (a) Subclass association (SA) matrix for the comparison between DLBCL-C and DLBCL-D data sets. Bonferroni-corrected p-values for the pairs of “C_3_ and D_2_” and “C_4_ and D_3_” were 0.002 and 0.002, respectively, (b) Survival prediction models were built using DLBCL-C and applied to DLBCL-D. Kaplan-Meier survival curves for the predicted groups in DLBCL-D are shown. Left: Prediction model was trained using all cases in DLBCL-C (n = 58), and tested on all cases in DLBCL-D (n = 129). Middle: Survival prediction using only cases from “matched” subclasses. Model was trained using C_3_ ∪ C_4_ samples (n = 25) and tested in D_2_ ∪ D_3_ (n = 61). The survival separation was better than that in the left panel in spite of having fewer samples. Right: survival prediction using only cases from “unmatched” subclasses. Model was trained using C_1_ ∪ C_2_ (n = 33) and tested in D_1_ ∪ D_4_ (n = 68). The numbers of events were 61, 22, and 39 for all (D_1_ ∪ D_2_ ∪ D_3_ ∪ D_4_), “matched” (D_2_ ∪ D_3_), and “unmatched” (D_1 _∪ D_4_) patients, respectively. p-values were calculated using the log-rank test. DFS: disease free survival.

Using hierarchical clustering, four candidate subclasses were defined in each data set (C_1_, C_2_, C_3_, and C_4_ in DLBCL-C, and D_1_, D_2_, D_3_, and D_4_ in DLBCL-D). There was no statistically significant difference in the survival curves among these subclasses (data not shown). SubMap identified two significantly corresponding pairs of subclasses between these two data sets, “C_3_ and D_2_” and “C_4_, and D_3_” (Figure 5a).

We next sought to determine whether we could improve the survival distinction between samples by restricting our consideration to the corresponding subclasses. Based on the sample labeling of poor prognosis in DLBCL-C defined in [14], we built a survival predictive model using a supervised learning method, k-nearest neighbor algorithm (k = 3, and 10 marker genes), and tested it in DLBCL-D (Figure 5b). When the model was built using only matched subclasses, C_3_ ∪ C_4_ (n = 25), and tested in their counterparts, D_2_ ∪ D_3_ (n = 61), the predictive model yielded better separation of survival than a model built using all of the samples (Figure 5b) in spite of having many fewer, nearly half, the number of samples to build and test the predictor. On the other hand, the predictor trained using unmatched subclasses, C_1_ ∪ C_2_ (n = 33) did not work on D_1_ ∪ D_4_ (n = 68) This suggests that the predictive survival signature common to both of the data sets is carried by the “corresponding” cases, which our method identified.

## Discussion

Genomic profiling is a powerful approach to discovering molecular disease subtypes. However, the uncovered subtypes may also reflect idiosyncrasies, technical variation, representational and measurement biases that limit the ability of the model and results to generalize to other studies or platforms. In this paper, we have shown how SubMap can address these issues and be an effective approach to recognizing subtypes across different data sets.

In the four examples presented above the method clearly outperforms several more traditional, alternative approaches such as, i) using supervised models (e.g., k-NN and SVM) to learn the subclasses obtained in one of the datasets and then predicting the subclass membership of the samples in the second set, ii) merging the two data sets and clustering samples to search for common subtypes, iii) associating the subtypes between the data sets by assessing the overlap of each subtype's marker genes in both data sets, or iv) creating an explicit model of subclassification in one data set, and evaluating it in another data set (e.g., clusterRepro). All of these alternative methods have serious limitations and do not always produce reproducible results. While two different datasets may claim to represent the same biological phenotype, they often contain different molecular subtypes or biased representations and samplings with different dynamic ranges, measurement biases, probe efficiencies etc. These differences violate the common underlying assumption, of these other approaches-that two instances of the data, such as the train and test set, are samplings from the same probability distribution.

SubMap associates the subclasses between datasets by using a more abstract, higher-level similarity measure (i.e., the enrichment score of ranked subclass markers). SubMap focuses on the concordant collective behavior of groups of genes rather than specific gene expression values, and does not rely on one particular data set to define a “model” like supervised learning methods. The result of this approach, which concurrently and bi-directionally evaluates similarity by treating the two input data sets equally within a self-contained unit, is that SubMap is able to provide solutions to the mapping problem that are more robust, more generalizable and more tolerant of the platform idiosyncrasies and biases.

SubMap performs marker gene selection multiple times. For example, in the case where data set A and B have 3 and 4 subclasses, respectively, it performs maker gene selection 7 times, 3 times in data set A and 4 times in data set B. We cannot assume the same number of statistically “significant” marker genes for all of them, and to compute the significance for each round of marker selection by, e.g., permutation testing is highly computationally intensive. The GSEA enrichment score (ES) is weighted by the correlation between gene expression signals and the subclass. Therefore, genes having small correlation with phenotype (i.e., less significant genes) show negligible contribution in the computation of the ES. For this reason, there is no need to set significance thresholds to select the marker genes. The fixed number of marker gene set only needs to include sufficient significant marker genes to produce a robust ES (see Figure S1).

Another important and useful property of SubMap is that it helps to identify an appropriate level of subclass granularity to select the best overall concordance. Typically in unsupervised clustering, the depth of splitting of the clustering solution is somewhat arbitrary. Even when a model selection method is used, there is the possibility of over-fitting to a dataset structure that represents batch effects or platform idiosyncrasies. As a consequence, a dominant structure in one data set might not necessarily show the best concordance with the other data set. A concrete example of this was shown in Example 3, where the second dominant splitting in Breast-B, rather than the first, showed the best concordance with Breast-A. The clustering of the subclass association matrix gives an indication of which candidate subclasses show the best concordance between the data sets.

The use of more robust and generalized subclasses enables the better utilization of microarray data and the construction of better classification models. For example, the possibility of mapping the corresponding classes across a large collection of cross-platform or cross-laboratory datasets provides a method to increases the number of available samples related to a given phenotype. This translates to a better signal to noise ratio and thus improves the selection of biomarkers and the training of more accurate, robust, and generalizable supervised classifiers. Moreover, SubMap can provide a more precise stratification of the data for the application of predictive models. In Example 4, our method identified subpopulations in two independent data sets, where a clinical outcome predictor is more accurate than one modeled by all the samples. This additional resolution might help explain why an outcome predictor works only on a subset of the population. The integration and utilization of multiple datasets, representing a wider span of the biological space of interest, can both increase the accuracy of models as described above and provide models that are more interpretable, realistic, and biologically meaningful.

The ability of SubMap to find common subtypes across data sets is, of course, dependent on the representation of the subtypes in those data sets, and the quality and granularity of their labeling. This is true whether the samples are labeled manually by, for example, a pathologist, or computationally by clustering. At a coarser granularity (i.e., smaller number of larger candidate subclasses), a biological subclass of interest might be missed. On the other hand, a finer granularity (i.e., larger number of smaller candidate subclasses) could yield an unbalanced comparison in selecting the marker genes. In addition, too fine a class splitting might yield multiple similar candidate subclasses, which might cause a failure to detect distinct marker genes resulting in lower sensitivity in detecting subclass association. However, we note here that, aside from the issue of increasing runtime, there is no *a priori* limit on the number of the candidate subclasses that can be associated as long as they have robust corresponding marker genes.

SubMap is an exploratory method, and the optimal (or biologically reasonable) number of the candidate subclasses would depend on the particular data sets with complex trade-offs. One possible approach is to define multiple possible candidate subclassifications in each data set, and evaluate the subclass association structure in all combinations of candidate subclassifications (Figure S4). To define an appropriate measure or statistic to evaluate such a search for optimal candidate subclasses will require further consideration and investigation.

In summary, SubMap is a method that provides more general molecular subclass identification and correspondence among a collection of microarray data sets. By allowing the integration of multiple data sets measured on different platforms, from different laboratories, it increases the possibility of extracting more meaningful biological subclasses and their corresponding biomarkers and classifiers. This has the potential to improve gene expression-based clinical classifiers where robustness, generalization, and reproducibility across platforms are paramount.

The entire methodology is implemented as the SubMap module of the GenePattern software and is freely available from http://www.broad.mit.edu/genepattern/.

## Materials and Methods

### Data preprocessing

We started from data sets that were already normalized for their respective study without any additional normalization procedure to account for different platform derivation. For the signal intensity data generated by one-channel oligonucleotide microarrays, Affymetrix's GeneChip, we applied a lower threshold of 20U and a upper threshold of 16,000U. For the log2 transformed ratio data generated by cDNA microarrays, we first removed genes whose values were missing in more than 5% of the samples, and then imputed the missing values for the rest of the genes using a k-nearest neighbor algorithm [15] (ImputeMissingValues.KNN, in the GenePattern software package, http://www.broad.mit.edu/genepattern/).

Before marker gene selection, we used following gene filtering. For the oligonucleotide array data, only genes exhibiting at least 3-fold differential expression and an absolute difference of at least 100 units across the samples in the experiment were included. For the cDNA array data, only genes with an absolute log2 ratio greater than one and whose difference in log2 ratio across all the samples in the data set was greater than one were included.

Before applying the SubMap, each microarray probe ID was converted into its corresponding HUGO gene symbol (http://www.gene.ucl.ac.uk/nomenclature/), and multiple probe data corresponding to a single gene symbol was averaged. The number of genes remaining for our analyses of multiple tissue types, DLBCL, breast cancer, and DLBCL (with survival data) data sets were 5565, 661, 1213, and 3795, respectively.

### Subclass mapping (SubMap)

The algorithm and pseudocode for SubMap is shown in Box S1. We describe the algorithm in more detail below.

#### i) Candidate subclasses

We emphasize here that SubMap is an approach to evaluate the relationship between otherwise determined subclass structures in independently generated data sets. Thus, any clustering method can be used to generate candidate subclass labels. Alternatively, biologically/clinically known phenotype information can be used as candidate subclass labels. For the specific examples described in the results section, we chose to use agglomerative hierarchical clustering to generate candidate subclass labels (Figure 1a) [16]. We used the Pearson correlation coefficient as a distance metric and the average linkage method from the dChip software (www.biostat.harvard.edu/complab/dchip). We determined subclass splitting by tracing from the root of the dendrogram. A subclass that contained at least 10% of the samples was considered as a possible candidate subclass. We set the maximum number of the candidate subclasses as four. Any other criteria could be used to define a candidate subclass.

#### ii) Similarity between candidate subclasses in each data set: enrichment score (ES)

Suppose data set A and B have *n_A_* and *n_B_* candidate subclasses, respectively. First, we take the top 100 differentially expressed genes between a candidate subclass (*CS*) and the rest of the candidate subclasses (*nonCS*) the data set A. Such marker genes are identified for each candidate subclass in A. In data set B, genes are ordered according to their differential expression with respect to *CS* vs. *nonCS*. To evaluate differential gene expression between *CS* and *nonCS*, any measure, e.g., *t*-statistic, can be used. In this study, we used the signal-to-noise ratio (SNR), (*μ_CS_*-*μ_nonCS_*)/(*σ_CS_*+*σ_nonCS_*), where *μ_i_* and *σ_i_* denote, respectively, the sample mean and sample standard deviation within class *CS* or *nonCS* [17]. Thus, every candidate subclass in A has a set of marker genes, and every candidate subclass in B has a gene list ranked by differential expression between the candidate subclass and the remaining candidate subclasses in B. Alternatively, other methods for gene selection and ranking such as neighborhood analysis [17], Significance Analysis of Microarrays [18], and the false discovery rate (FDR)-based approaches [19] can easily be used.

For every pair-wise combination of *n_A_* marker gene sets and *n_B_* gene rankings, class similarity is measured by calculating a gene set enrichment score (ES) and estimating its significance as previously described in [3]. Briefly, in each ranked gene list for B*_j_* (*j*∈{1,…,*n_B_*}), a marker gene set of a candidate subclass A*_i_* (*i*∈{1,…,*n_A_*}) is identified, and its enrichment or over-representation at the top of the list is measured by computing a ES (observed ES_A*i*B*j*_). The ES is essentially a Kolmogorov-Smirnov score weighted by the SNR.

We recompute the ES_A*i*B*j*_ for 1000 random assignments of the subclass labels in B*_j_* to generate a null distribution for ES_A*i*B*j*_. We sort the resulting ES scores in the null distribution, and estimate the significance of the observed ES_A*i*B*j*_ from its rank in the list as a nominal p-value (p_A*i*B*j*_). We repeat this process by interchanging the role of the data sets A and B. Thus, we compute p_A*i*B*j*_ and p_B*j*A*i*_.

#### iii) Significance of association between candidate subclasses

We next sought a summary statistic for p_A*i*B*j*_ and p_B*j*A*i*_ in order to obtain combined mutual marker gene enrichment information as a measure of similarity of subclasses in the two input data sets. We use the Fisher inverse chi-square statistic, *F_ij_ = -2*(log(*p_AiBj_*)*+*log(*p_BjAi_*)) to summarize the two p-values (Figure 1b) [4], [20]. The significance of *F_ij_* is estimated as a one-sided nominal p-value in a null distribution for *F_ij_* generated by randomly picking one rank p-value from the null distribution for each of ES_A*i*B*j*_ and ES_B*j*A*i*_ and evaluating their Fisher statistic 10,000 times.

#### iv) Subclass association (SA) matrix

We now have n_A_*n_B_ nominal p-values for all *F_ij_* (*i*∈{1,…,*n_A_*}, *j*∈{1,…,*n_B_*}). To correct for multiple hypothesis testing, a Bonferroni correction is applied. Alternatively, for cases with a larger number of candidate subclasses, we can estimate a FDR by multiplying each nominal p-value by n_A_*n_B_ and dividing by the rank of the nominal p-value. Using these corrected p-values, we obtain a subclass association (SA) matrix, which represents the global association structure of the candidate subclasses in the two independent data sets. By applying two-way clustering on the SA matrix (we used hierarchical clustering), it becomes possible to evaluate whether there are common subclasses that exist in both data sets.

### Prediction analysis

For prediction analysis, we used k-nearest neighbor (k-NN) and support vector machine (SVM) modules from the GenePattern software. For k-NN, briefly, genes in the training data set were rank-ordered by the SNR. Using the selected marker genes, prediction was performed based upon a majority vote of the class membership of its k nearest neighbors in the training set weighted by the reciprocal of the cosine distance. The k and the number of marker genes were chosen so as to minimize the leave-one-out cross-validation (LOOCV) error rate in the training set.

In Example 4, the prediction models were built based on LOOCV. Fractional use of each gene in the LOOCV models was calculated, and top 5 most frequently used genes were used for class prediction in the test data set (Figure S3).

### Survival analysis

Kaplan-Meir analysis and the log-rank test were performed using GenePattern SurvivalCurve and SurvivalDifference modules, respectively.

## Supporting Information

Table S1 (0.02 MB XLS)

Table S2 (0.02 MB XLS)

Table S3 (0.01 MB XLS)

Figure S1 Effect of the number of marker genes on the result of SubMap.
(0.10 MB TIF)

Figure S2 Two breast cancer data sets, Breast-A and Breast-B, are directly merged and clustered. (a) Each column was normalized by subtracting the column mean and divided by the column SD before clustering. (b) Gene expression data are converted to their rank in each column, and clustering was performed using the rank to compute the distance. Pearson correlation and the average linkage method were used for the hierarchical clustering.
(0.08 MB TIF)

Figure S3 Genes used for the survival prediction in Example 4. Leave-one-out cross-validation (LOOCV) is performed using C_3_ ∪ C_4_ in DLBCL-C as described in the Method section. Bar indicates fractional use of each gene in LOOCV models. Box indicates top 5 most frequently used genes in LOOCV that is used for prediction in D_2_ ∪ D_3_ in DLBCL-D. Genes shown in red are also included in a prediction model built using all samples.
(0.05 MB TIF)

Figure S4 Effect of granularity of the candidate subclasses on SubMap result. In Breast-A and Breast-B data sets in Example 3, the finest granularity (i.e., the largest number of candidate subclasses) was defined as subclasses having at least 10% of the cohort. Each subclass was labeled by “number of subclasses”-“data set”-“subclass number”. In Breast-A, we defined sets of two (2-A_1_ and 2-A_2_) and three (3-A_1_, 3-A_2_, and 3-A_3_) candidate subclasses. In Breast-B, we defined sets of two (2-B_1_ and 2-B_2_), four (4-B_1_, 4-B_2_, 4-B_3_, and 4-B_4_), and six (6-B_1_, 6-B_2_, 6-B_3_, 6-B_4_, 6-B_5_, and 6-B_6_) candidate subclasses. SubMap was performed on all combinations of sets of the candidate subclasses. When the coarsest granularity (i.e., the smallest number of candidate subclasses) was assumed in Breast-B, we observed no significant subclass association (left heatmaps). When finer granularity was assumed for Breast-B (middle heatmaps), significant “two-class” correspondence started to appear, indicating the coarsest granularity in Breast-B was not appropriate to find significant subclass association. The finest granularity for Breast-A derived more significant associations (middle bottom heatmap). When the finest granularity was assumed in Breast-B, a small fraction of samples (6-B_6_) showed no association with any subclasses in Breast-A (right heatmaps), suggesting that this is too fine a granularity yielding weaker marker genes and lower sensitivity to capture a counterpart of 6-B_6_.
(0.13 MB TIF)

Box S1 Algorithm to generate a SA matrix.
(0.29 MB DOC)
