# Supplementary figures and images for: Subclass Mapping: Identifying Common Subtypes in Independent Disease Data Sets
Source: PLoS One. 2007 Nov 21;2(11):e1195. doi: 10.1371/journal.pone.0001195 (PMC2065909; doi:10.1371/journal.pone.0001195)

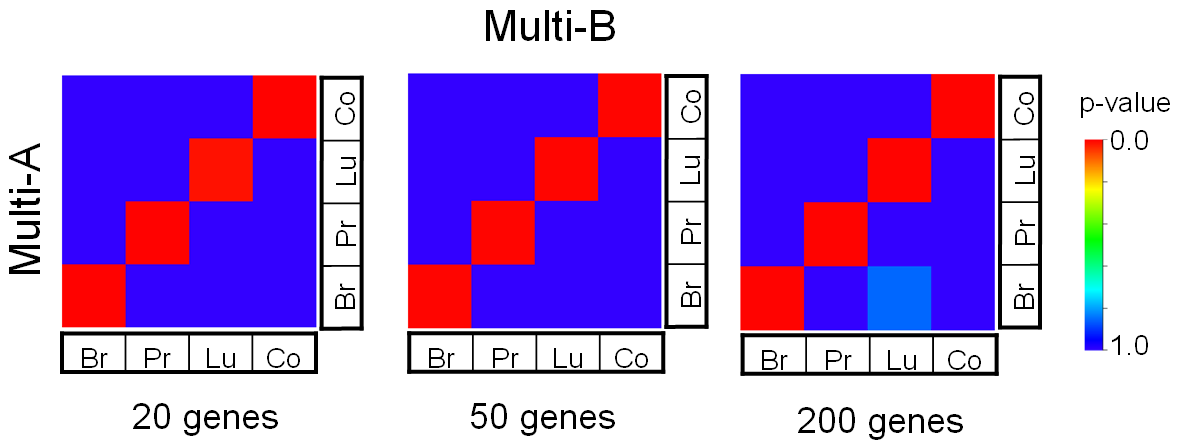

Supplement: Figure S1 — Effect of the number of marker genes on the result of SubMap. (0.10 MB TIF) [file pone.0001195.s004.tif]

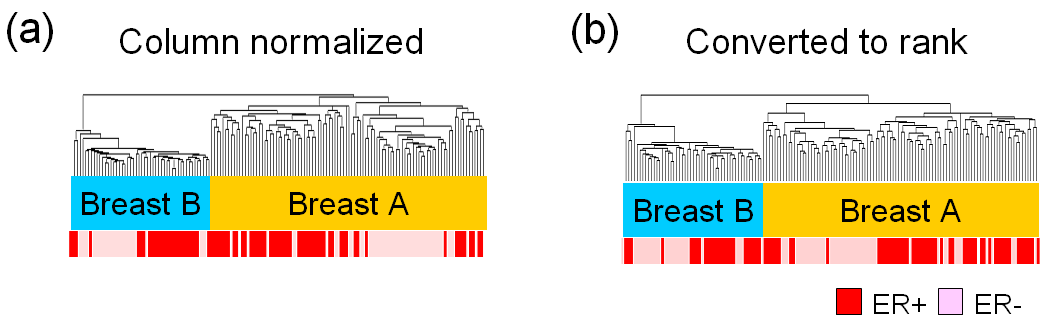

Supplement: Figure S2 — Two breast cancer data sets, Breast-A and Breast-B, are directly merged and clustered. (a) Each column was normalized by subtracting the column mean and divided by the column SD before clustering. (b) Gene expression data are converted to their rank in each column, and clustering was performed using the rank to compute the distance. Pearson correlation and the average linkage method were used for the hierarchical clustering. (0.08 MB TIF) [file pone.0001195.s005.tif]

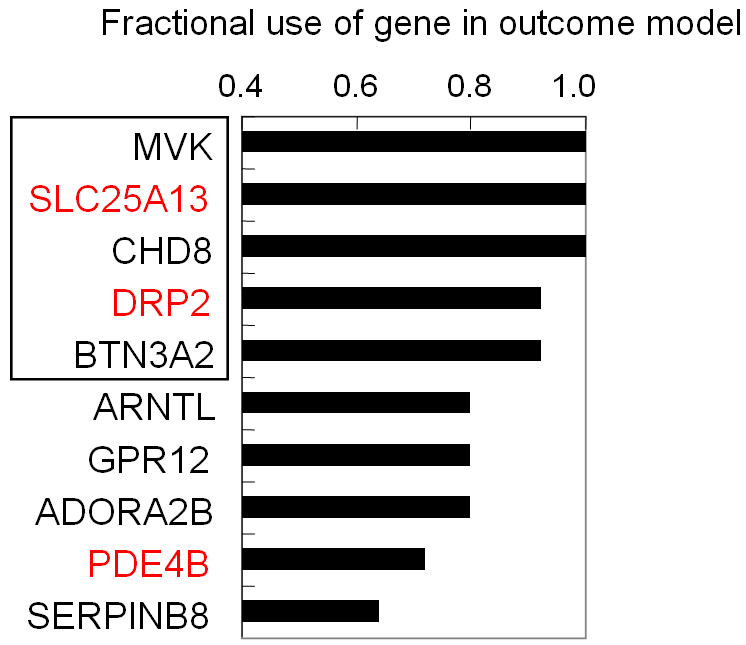

Supplement: Figure S3 — Genes used for the survival prediction in Example 4. Leave-one-out cross-validation (LOOCV) is performed using C3 ∪ C4 in DLBCL-C as described in the Method section. Bar indicates fractional use of each gene in LOOCV models. Box indicates top 5 most frequently used genes in LOOCV that is used for prediction in D2 ∪ D3 in DLBCL-D. Genes shown in red are also included in a prediction model built using all samples. (0.05 MB TIF) [file pone.0001195.s006.tif]

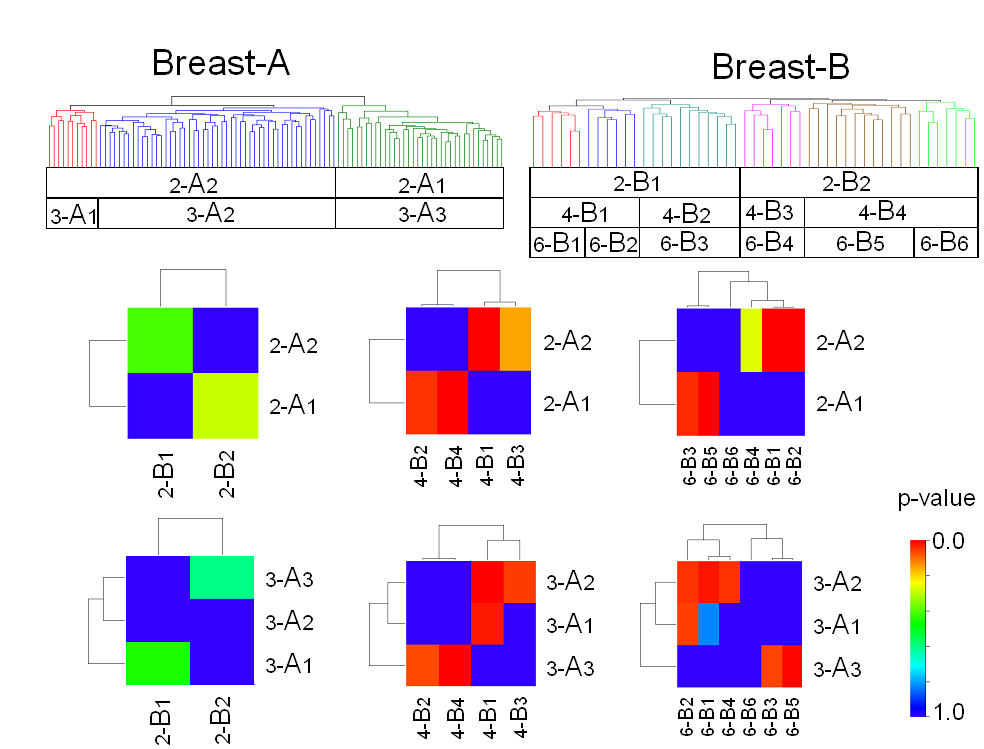

Supplement: Figure S4 — Effect of granularity of the candidate subclasses on SubMap result. In Breast-A and Breast-B data sets in Example 3, the finest granularity (i.e., the largest number of candidate subclasses) was defined as subclasses having at least 10% of the cohort. Each subclass was labeled by “number of subclasses”-“data set”-“subclass number”. In Breast-A, we defined sets of two (2-A1 and 2-A2) and three (3-A1, 3-A2, and 3-A3) candidate subclasses. In Breast-B, we defined sets of two (2-B1 and 2-B2), four (4-B1, 4-B2, 4-B3, and 4-B4), and six (6-B1, 6-B2, 6-B3, 6-B4, 6-B5, and 6-B6) candidate subclasses. SubMap was performed on all combinations of sets of the candidate subclasses. When the coarsest granularity (i.e., the smallest number of candidate subclasses) was assumed in Breast-B, we observed no significant subclass association (left heatmaps). When finer granularity was assumed for Breast-B (middle heatmaps), significant “two-class” correspondence started to appear, indicating the coarsest granularity in Breast-B was not appropriate to find significant subclass association. The finest granularity for Breast-A derived more significant associations (middle bottom heatmap). When the finest granularity was assumed in Breast-B, a small fraction of samples (6-B6) showed no association with any subclasses in Breast-A (right heatmaps), suggesting that this is too fine a granularity yielding weaker marker genes and lower sensitivity to capture a counterpart of 6-B6. (0.13 MB TIF) [file pone.0001195.s007.tif]
