# Supplementary material for: Subclass Mapping: Identifying Common Subtypes in Independent Disease Data Sets
Source: PLoS One. 2007 Nov 21;2(11):e1195. doi: 10.1371/journal.pone.0001195 (PMC2065909; doi:10.1371/journal.pone.0001195)
Supplement: Box S1 — Algorithm to generate a SA matrix. (0.29 MB DOC) [file pone.0001195.s008.doc]

(1) Load gene expression data sets: A, B

candidate subclass/phenotype labels: clsA, clsB

(2) Set observed ES matrix: ES_AonB_[n_A_,n_B_]

permutation ES matrices: pES_AonB_[n_A_,n_B_,nperm]

nominal-p matrix: nomP_AonB_[n_A_,n_B_,nperm+1]

(3) FOR i=1 to n_A_

select marker gene of A[i]

FOR j=1 to n_B_

generate gene ranking by B[j]
    calculate ES_AonB_[i,j] by mapping marker genes of A[i]

on gene ranking by B[j]

generate nperm random permutations of clsB

compute pES_AonB_[i,j,nperm] for permutations of clsB

compute nomP_AonB_[i,j,nperm+1] using rank of each ES

ENDFOR

ENDFOR

(4) Repeat (2) and (3) by switching A and B

(5) Set Fisher statistics matrices: fisher[n_A_,n_B_,npermFisher+1]

SA matrix: SA[n_A_,n_B_]

(6) FOR i=1 to n_A_

FOR j=1 to n_B_

fisher[i,j,1]=-log(nomP_AonB_[i,j,1])-log(nomP_BonA_[j,i,1])

FOR k=2 to npermFisher+1

p1<-one random sample from nomP_AonB_[i,j,2:nperm+1]

p2<-one random sample from nomP_BonA_[j,i,2:nperm+1]

fisher[i,j,k]=-log(p1)-log(p2)

ENDFOR

SA[i,j]<-nominal-p of fisher[i,j,1]

in fisher[i,j,npermFisher+1]

ENDFOR

ENDFOR

(7) SA 🡨 SA*(n_A_*n_B_) (Bonferroni correction)

SA 🡨 SA*(n_A_*n_B_)/(rank of nominal-p) (FDR correction)
